# Mobilization of Nuclear Copper by Green Tea Polyphenol Epicatechin-3-Gallate and Subsequent Prooxidant Breakage of Cellular DNA: Implications for Cancer Chemotherapy

**DOI:** 10.3390/ijms18010034

**Published:** 2016-12-26

**Authors:** Mohd Farhan, Mohammad Oves, Sandesh Chibber, Sheikh Mumtaz Hadi, Aamir Ahmad

**Affiliations:** 1 Department of Biochemistry, Faculty of Life Sciences, AMU, Aligarh 202001, India; farhan@mohdfarhan.com (M.F.); sandeshchibber@gmail.com (S.C.); 2 Center of Excellence in Environmental Studies, King Abdulaziz University, Jeddah 21589, Saudi Arabia; owais.micro@gmail.com; 3 Oncologic Sciences, Mitchell Cancer Institute, University of South Alabama, 1660 Springhill Avenue, Mobile, AL 36604-1405, USA

**Keywords:** epicatechin-3-gallate, copper, DNA damage, comet assay, reactive oxygen species, prooxidant, anticancer

## Abstract

Epidemiological as well as experimental evidence exists in support of chemopreventive and anticancer properties of green tea and its constituents. The gallocatechin, epicatechin-3-gallate is a major polyphenol present in green tea, shown responsible for these effects. Plant-derived polyphenolic compounds are established natural antioxidants which are capable of catalyzing oxidative DNA degradation of cellular DNA, alone as well as in the presence of transition metal ions, such as copper. Here we present evidence to support that, similar to various other polyphenoic compounds, epicatechin-3-gallate also causes oxidative degradation of cellular DNA. Single cell alkaline gel electrophoresis (Comet assay) was used to assess DNA breakage in lymphocytes that were exposed to various concentrations of epicatechin-3-gallate. Inhibition of DNA breakage in the presence of scavengers of reactive oxygen species (ROS) suggested involvement of ROS generation. Addition of neocuproine (a cell membrane permeable Cu(I) chelator) inhibited DNA degradation, dose-dependently, in intact lymphocytes. In contrast, bathocuproine, which does not permeate cell membrane, was observed to be ineffective. We further show that epicatechin-3-gallate degrades DNA in cell nuclei, which can also be inhibited by neocuproine, suggesting mobilization of nuclear copper in this reaction as well. Our results are indicative of ROS generation, possibly through mobilization of endogenous copper ions, and support our long-standing hypothesis of a prooxidant activity of plant-derived polyphenols as a mechanism for their documented anticancer properties.

## 1. Introduction

Plant-derived polyphenols possess several pharmacological properties, mechanisms of which are not clearly understood. These polyphenols, represented by curcuminoids (such as curcumin), flavonoids, tannins, gallocatechins (such as epigallocatechin-3-gallate), stilbenes (such as resveratrol) etc., are natural antioxidants often recognized for antiviral and antitumour properties [1,2]. Independent studies have reported that gallocatechins found in green tea, such as tannic acid, gallic acid, epigallocatechin, epicatechin-3-gallate and epigallocatechin-3-gallate induce apoptosis in several cancer cell lines [3,4]. Likewise, curcumin [5], from turmeric, and resveratrol [6], from red grapes and red wine, also induce apoptosis in cancer cells. Green tea consumption has been suggested to reduce risk of various cancers such as bladder, prostate, oesophagus and stomach cancer [4]. Particularly interesting is the observation that epigallocatechin-3-gallate induces internucleosomal DNA fragmentation in the human epidermoid and prostate cancer cells as well as the, mouse lymphoma cells, without affecting the normal epidermal keratinocytes [4]. Gallic acid also exhibited cytotoxicity against many cancer cell lines, but not against primary cultured rat hepatocytes and macrophages [3]. Resveratrol can induce apoptotic cell death in HL60 human leukaemia cells, but not in normal peripheral blood lymphocytes [6]. Previous work in our laboratory has established that plant-derived polyphenols such as flavonoids [7], tannic acid and its structural constituent gallic acid [8], curcumin [9], gallocatechins [10] and resveratrol [11] are capable of causing oxidative strand breakage in DNA, both alone as well as in the presence of transition metal ions such as copper. Copper is an important metal ion present in chromatin that closely associates with DNA bases, particularly guanine [12]. Copper also happens to be the most redox active metal ion in the cells. Most of plant-derived polyphenols exhibit antioxidant as well as prooxidant properties [3,7], and investigations in our lab over past many years have revealed that the prooxidant action of plant-derived polyphenolics may be an important mechanism of their anticancer properties [13]. In addition, a few recent studies have confirmed that elevating the generation of ROS over a critical threshold, through lowering of antioxidant defences, can result in selective killing of cancer cells, through mobilization of endogenous copper ions, possibly the copper that is bound to chromatin, and the consequent prooxidant action [14,15].

**Figure 1:**
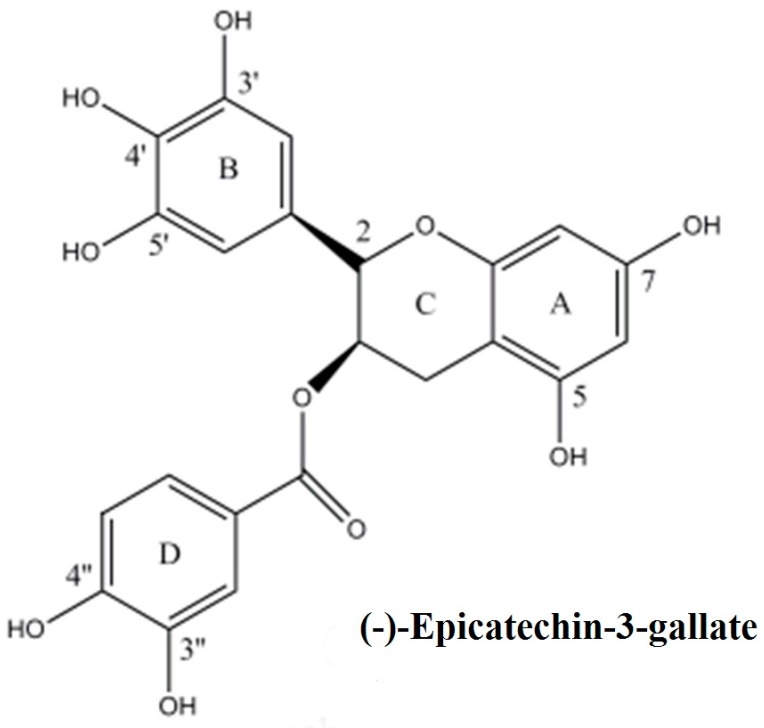
Chemical structure of (−)-epicatechin-3-gallate.

Through alkaline single cell gel electrophoresis (Comet assay) on a cellular system of peripheral lymphocytes, that were isolated from human blood, here we confirm that epicatechin-3-gallate is capable of causing DNA degradation in cells, in the presence of Cu(II). Further, we also show that the observed DNA degradation in lymphocytes can be attenuated by scavengers of ROS as well as by neocuproine, an agent that specifically sequesters Cu(I). These observations are suggestive of DNA breakage, brought about ROS generated through the polyphenols-induced reduction of Cu(II) to Cu(I) [16]. The chemical structure of epicatechin-3-gallate is given in Figure 1.

## 2. Results

### 2.1. Cellular DNA Breakage by Epicatechin-3-Gallate in Intact Cells and Permeabilized Cells as Measured by Comet Assay

**Figure 2:**
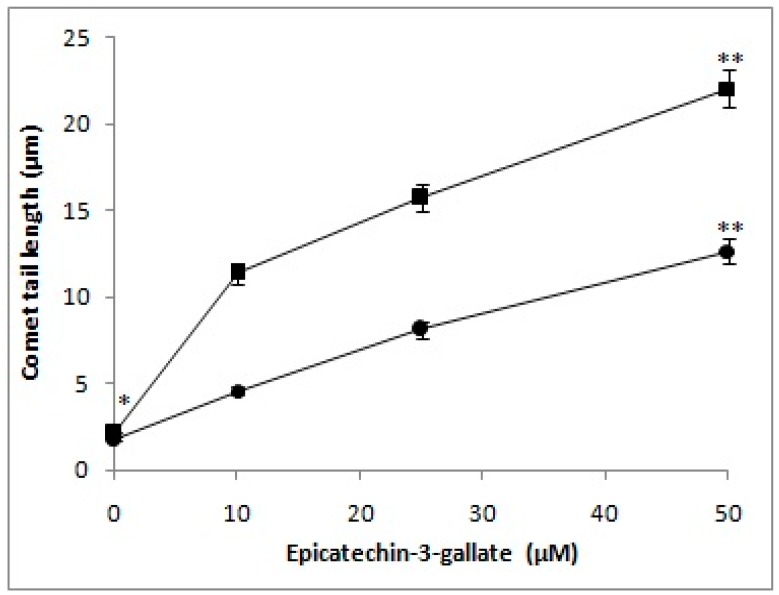
A comparison of cellular DNA breakage induced by epicatechin-3-gallate in intact cells (filled circles) and permeabilized cells (filled square) as a function of comet tail lengths. Values reported are mean ± SEM of three independent experiments. ** *p <* 0.01, compared to control (* 0 μM epicatechin-3-gallate).

**Figure 3:**
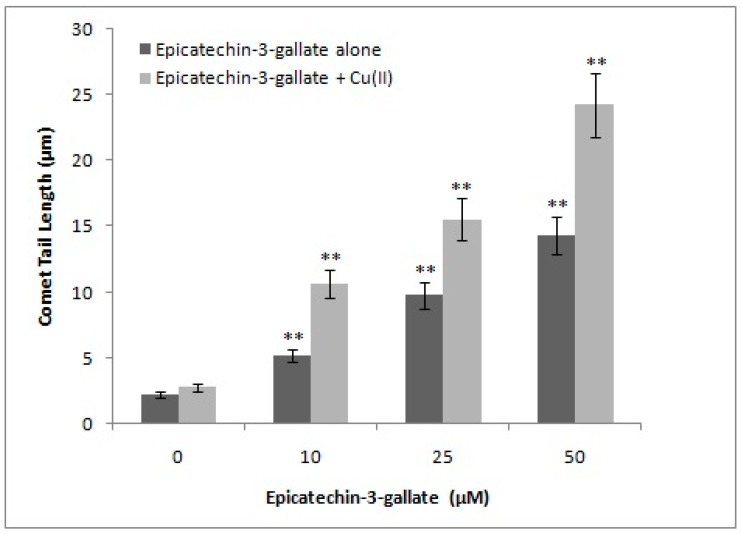
Cellular DNA breakage by epicatechin-3-gallate in human peripheral lymphocytes in the absence and presence of Cu(II). Comet tail length (µm) plotted as a function of increasing concentrations of epicatechin-3-gallate (0–50 µM) in the absence and presence of 50 µM Cu(II). All points represent mean of three independent experiments. Error bars denote mean ± SEM. ** *p <* 0.05, compared to control (in the absence of epicatechin-3-gallate).

Previous work in our laboratory has established that various classes of polyphenols are capable of degrading cellular DNA, when incubated with human peripheral lymphocytes, and that such degradation can be analyzed by Comet assay [17]. In the present study, increasing concentrations of epicatechin-3-gallate (10, 25, and 50 µM) were tested for their ability to induce DNA breakage in isolated lymphocytes. As seen in Figure 2, we observed a dose-dependent increase in DNA breakage, which can be visualized by increasing comet tail lengths that are indicative of significant breakage of cellular DNA. As per our hypothesis, polyphenols mobilize chromatin-bound copper, resulting in cellular DNA breakage. We, therefore, tested the effect of epicatechin-3-gallate treatment on permeabilized lymphocytes as well (Figure 2). Permeabilized lymphocytes allow direct interaction of epicatechin-3-gallate/polyphenols with cell nuclei. Because of the direct interaction, considerably greater DNA breakage should be observed in permeabilized lymphocytes, as compared to the intact cells. As observed in Figure 2, this was indeed found to be true. We also observed that the rate of comet tail formation induced by epicatechin-3-gallate was greater in the case of permeabilized lymphocytes, relative to intact cells, suggesting the ability of epicatechin-3-gallate to interact efficiently with the nuclei in a permeabilized system. We also tested, by standard comet assay, the ability of epicatechin-3-gallate to induce DNA strand breaks in cellular system represented by human peripheral lymphocytes, in the absence as well as the presence of Cu(II). As seen in Figure 3, epicatechin-3-gallate caused breakage of cellular DNA. The extent breakage was visibly enhanced by the presence of copper. Cu(II) (50 µM) controls did not exhibit significant DNA breakage, and were similar to untreated lymphocytes (see Figure S1 for Single cell gel electrophoresis of human peripheral lymphocytes showing Comets (100×) after treatment with varying concentrations of Epicatechin-3-gallate and Cu(II) (50 µM)).

### 2.2. Effect of Reactive Oxygen Scavengers on the Epicatechin-3-Gallate Induced Cellular DNA Breakage in Permeabilized Cells

In order to confirm a role of ROS generation in the epicatechin-3-gallate-induced lymphocyte DNA breakage, we investigated the effect of selective scavengers of ROS on comet tail length [18,19]. Table 1 provides results of the experiment where an effect of catalase, SOD, and thiourea were tested on epicatechin-3-gallate-induced DNA degradation in permeabilized lymphocytes. Catalase removes H_2_O_2_, SOD removes superoxide, and thiourea removes hydroxyl radicals. From the results, we conclude that epicatechin-3-gallate-induced cellular DNA degradation is a result of ROS generation. The hydroxyl radical as well as the superoxide anion are proximal DNA damaging radicals. Further, superoxide anions are well known to result in spontaneous production of H_2_O_2_ that leads to the generation of hydroxyl radicals, through a process called fenton reaction, which involves re-oxidation of reduced transition metals, such as copper [20].

### 2.3. Effect of Metal-Specific Chelators on the Epicatechin-3-Gallate Induced Cellular DNA Breakage in Intact Cells and Permeabilized Cells

In the experiment shown in Table 2, we used various metal chelators, specific for copper, iron and zinc, to study the role of these individual metal ions in epicatechin-3-gallate-induced DNA degradation in intact cells as well as in permeabilized cells. In intact cells, neocuproine inhibited DNA degradation. Neocuproine is a cell membrane-permeable Cu(I)-specific chelator. However, no inhibition was observed when bathocuproine (a water soluble membrane-impermeable analogue of neocuproine) was used. Similarly, desferrioxamine mesylate (Fe(II)-specific chelator), and histidine (a zinc-specific chelator) also failed to afford inhibition. In contrast, both neocuproine and bathocuproine were found to be effective in permeabilized cells, whereas iron and zinc chelators continued to be ineffective, even in permeabilized cells. Bathocuproine, impermeable to cell membrane, could still traverse through permeabilized cells, and directly interact with cell nuclei. These results suggest that epicatechin-3-gallate mobilizes chromatin-bound copper, leading to an oxidative DNA breakage.

### 2.4. Cleavage of Plasmid pBR322 DNA by Epicatechin-3-Gallate

**Figure 4:**
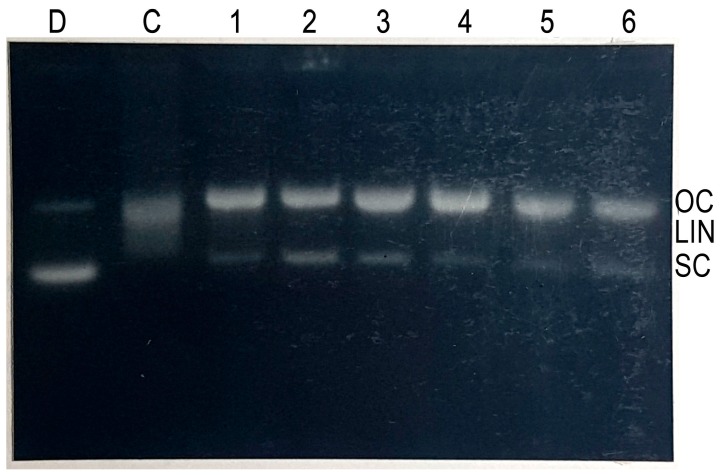
Agarose gel electrophoresis of pBR322 plasmid DNA treated with epicatechin-3-gallate and Cu(II). Reaction was performed in dark for 2 h at 37 °C. Lane D: pBR322 DNA alone; lane C: pBR322 DNA + Cu(II) 30 µM; lanes 1, 2, 3: pBR322 DNA + epicatechin-3-gallate (25, 50, 75 µM, respectively); lanes 4, 5, 6: pBR322 DNA + Cu(II) 50 µM + epicatechin-3-gallate (25, 50, 75 µM, respectively).

In order to examine the efficacy of DNA cleavage by epicatechin-3-gallate-Cu(II) system , we tested the ability of epicatechin-3-gallate to cause cleavage of supercoiled plasmid pBR322 DNA in the presence of copper ions. As seen in Figure 4, whereas epicatechin-3-gallate alone showed only minor DNA cleavage, addition of copper resulted in much enhanced DNA cleavage. This demonstrates that epicatechin-3-gallate is capable of cleaving plasmid DNA in the presence of copper ions.

### 2.5. Cu(II) Mediated Formation of ROS: DCFH-DA Assay

**Figure 5:**
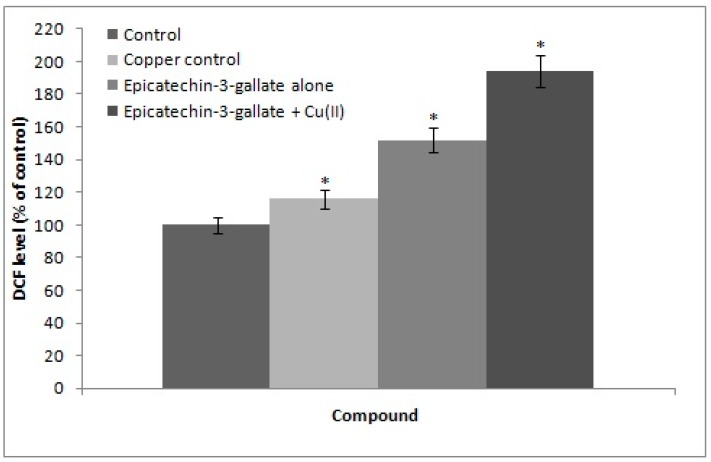
Intracellular ROS generation as determined by DCFH-DA assay on epicatechin-3-gallate alone and epicatechin-3-gallate + Cu(II) treated lymphocytes. Fluorescence intensity was recorded at 528 nm using excitation wavelength of 485 nm. Results are mean ± SEM of three independent experiments. * *p <* 0.05, compared to control cells.

We determined intracellular ROS generation using DCFH assay as described in the materials and methods section. DCFH-DA (2,7-dichlorodihydrofluorescein diacetate) is a non-fluorescent probe that enters cells where it gets converted to DCFH by intracellular esterases. In the presence of ROS, DCFH is rapidly oxidized to fluorescent DCF. As seen in Figure 5, in the absence of copper, epicatechin-3-gallate alone generates some ROS. However, in the presence of 50 µM added copper, a much more enhanced generation of ROS by epicatechin-3-gallate was evident.

### 2.6. H_2_O_2_ Generation by Epicatechin-3-Gallate in the Incubation Medium

**Figure 6:**
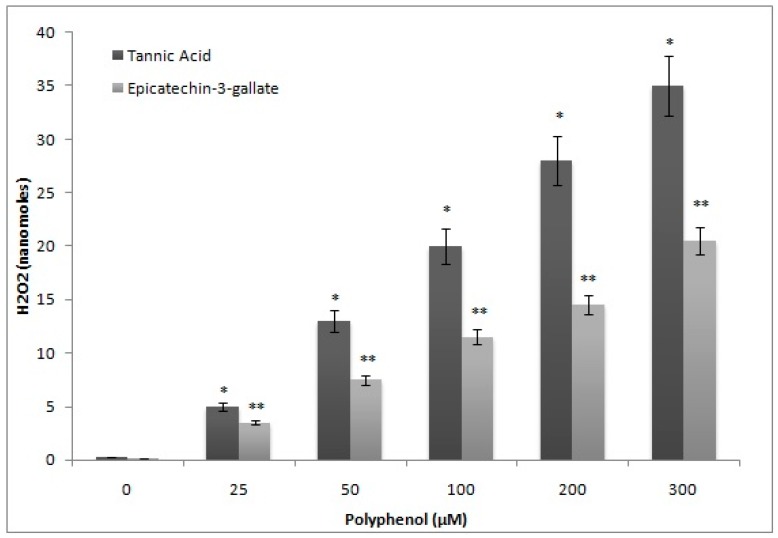
A comparison of the rate of H_2_O_2_ formation by tannic acid and epicatechin-3-gallate in the incubation medium as determined by FOX assay. *p <* 0.01, when ** values were compared to * values.

**Figure 7:**
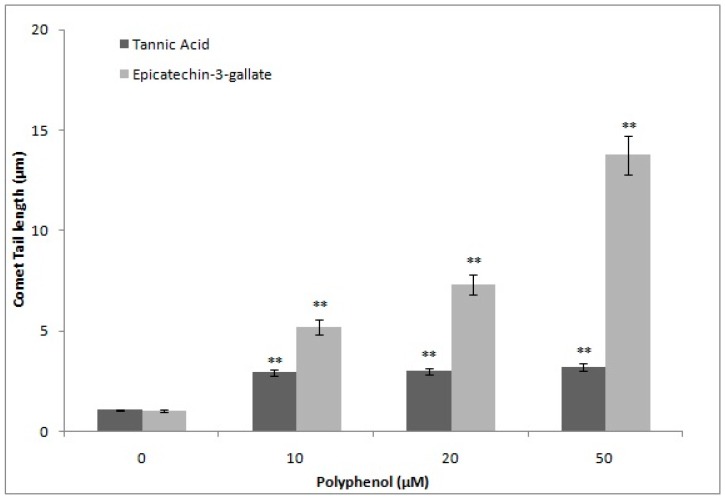
Comparison of DNA breakage induced by tannic acid and epicatechin-3-gallate in human peripheral lymphocytes as a function of comet tail lengths. ** *p <* 0.01, compared to respective untreated control.

Polyphenols auto-oxidize in cell culture media resulting in generation of H_2_O_2_ and quinone, which enter nuclei and cause damage to various macromolecules [21]. This leads to generation of ROS that also contribute towards cellular DNA breakage. In order to exclude this possibility, we determined the formation of H_2_O_2_, after epicatechin-3-gallate treatment, and compared it with tannic acid, a well-known generator of H_2_O_2_ [22]. As shown in Figure 6, the rate of formation of H_2_O_2_ by tannic acid in the incubation medium was considerably greater whereas that by epicatechin-3-gallate was significantly less. Further, we also compared the comet tail lengths as a function of increasing epicatechin-3-gallate and tannic acid concentrations. Further, as seen in Figure 7, whereas treatment with epicatechin-3-gallate dose-dependently resulted insignificant comet tail formation, treatment with tannic acid was not effective. These results indicate that with increasing concentrations of polyphenols (tannic acid and ECG) the H_2_O_2_ formation by tannic acid is considerably greater than ECG. On the other hand, DNA degradation (comet tail length) is consistently greater by ECG whereas that with tannic acid remains considerably lower.

### 2.7. Determination of TBARS as a Measure of Oxidative Stress in Lymphocyte Nuclei by Epicatechin-3-Gallate in the Presence of Neocuproine and Thiourea

**Figure 8:**
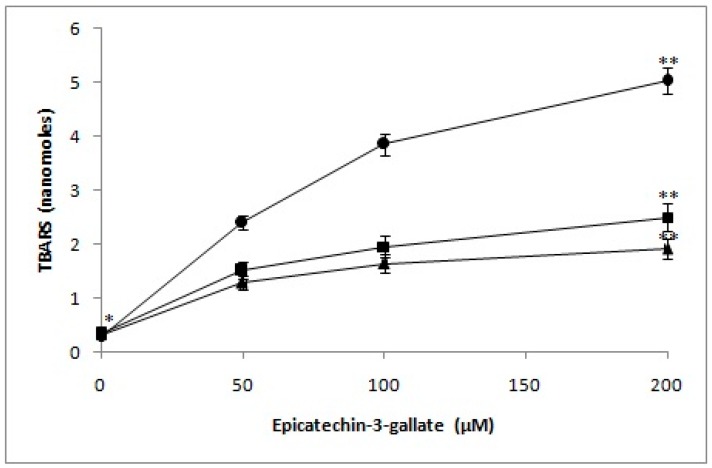
Effect of neocuproine and thiourea on TBARS production by epicatechin-3-gallate. Epicatechin-3-gallate alone (circle), Epicatechin-3-gallate + neocuproine (1 mM) (square) and Epicatechin-3-gallate + thiourea (1 mM) (triangle). The lymphocyte nuclei were pre-incubated neocuproine or thiourea at 37 °C for 30 min, followed by exposure to indicated doses of epicatechin-3-gallatefor 1 h. Values reported are mean ± SEM of three independent experiments. ** *p <* 0.05, compared to control (* 0 μM epicatechin-3-galate).

As per our hypothesis, DNA breakage observed in the nuclei of lymphocytes is a result of hydroxyl radicals generation, as well as other ROS in situ. Damage by oxygen radicals to deoxyribose or DNA is considered to give rise to TBA-reactive material [23,24]. Therefore, we determined the formation of TBARS as a measure of oxidative stress in lymphocyte nuclei, when exposed to increasing epicatechin-3-gallate concentrations. We also evaluated the effect of pre-incubating nuclei with neocuproine and thiourea. As seen in Figure 8, a dose-dependent increase in formation of TBA reactive substance in lymphocyte’s nuclei was induced by epicatechin-3-gallate. However, neocuproine and thiourea resulted in considerable decrease in the rate of TBARS formation. These results are indicative of inhibition of cellular DNA breakage in nuclei by Cu(I) chelation and scavenging of ROS.

### 2.8. Epicatechin-3-Gallate Causes Inhibition of Cell Growth in Breast Cancer Cells

**Figure 9:**
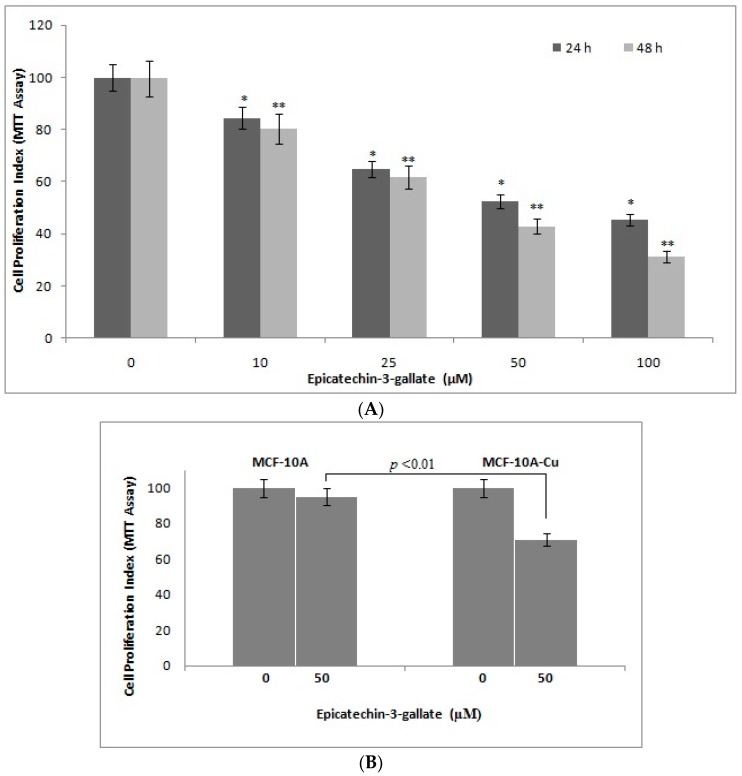
(**A**)The effects of epicatechin-3-gallate on the growth of MDA-MB-468 breast cancer cells as detected by MTT assay (metabolic response). The cells were incubated with indicated concentrations of epicatechin-3-gallate for 24 and 48 h, the results are expressed relative to control (vehicle-treated) cells; (**B**) MCF10A (normal breast epithelial cells) and MCF10A + Cu (MCF-10A cells cultured in copper-enriched medium) were treated with either vehicle (0 µM) or 50 µM epicatechin-3-gallate for 72 h. All results are expressed as percentage of control mean ± SEM of three independent experiments. * *p <* 0.05 and ** *p <* 0.01, compared to respective untreated control (0 µM epicatechin-3-gallate).

In Figure 2 and Figure 3, we showed strand breaks in cellular DNA by epicatechin-3-gallate. Next, we studied the effects of epicatechin-3-gallate on proliferative potential of human breast cancer cells MDA-MB-468. As seen in Figure 9A, we observed a dose-dependent inhibition of proliferation of MDA-MB-468 cells by epicatechin-3-gallate. These results are in agreement with the results from cellular DNA breakage experiment. We also observed (Figure 9B) that the normal breast epithelial cells, MCF-10A, were evidently resistant to epicatechin-3-gallate treatment but their continued culture in copper-rich medium resulted in sensitization to epicatechin-3-gallate action. These results support our earlier published results [25] that also focused on plant-derived polyphenolic compounds.

## 3. Discussion

The results from this present study suggest that, similar to several other classes of plant-derived polyphenols, the gallocatechin epicatechin-3-gallate also causes (i) cellular DNA degradation in the absence as well as presence of copper ions; (ii) such DNA breakage involves generation of ROS; and (iii) epicatechin-3-gallate induces DNA breakage in cell nuclei through a mechanism that involves mobilization of nuclear copper. Thus, essentially the conclusion of the above result is that epicatechin-3-gallate is capable of mobilizing endogenous copper both in normal as well as cancer cells. Since most cancer cells have elevated levels of copper that are preferentially attacked as compared with normal cells. We have earlier shown that in the case of plant polyphenols apigenin and luteolin the concentration required for cancer cells can be as low as 20 µM where as for normal cells it is considerably higher [25]. In the last twenty-five years, it has been recognized that plant derived polyphenols, such as resveratrol, tea catechins and curcuminoids possess anticancer properties. Such anticancer activity has been primarily attributed to their antioxidant behavior. However, it has also been demonstrated that most of these molecules induce cell death via apoptosis, suppressing anti apoptotic pathways and through the modulation of a number of proteins implicated in sustaining the growth of cancer cells [26]. Nevertheless, there is no single unifying mechanism supporting their anticancer effects both in vitro and in vivo. Most importantly, researchers have not yet identified the main mechanism, if any, that underlies the preferential cancer-specific action of these agents.

Our hypothesis explains the cancer cell-specific activity by suggesting a role of increased copper levels in cancer cells, which facilitates electron transfer between copper and plant-derived polyphenols, leading to ROS generation, namely superoxide and hydroxyl radicals, in the immediate vicinity of cellular DNA. It is, therefore, plausible that, at high concentrations, plant-derived polyphenols can also be toxic to normal cells. Our previous publications support this observation [16,18,19]. Towards this end, it should be noted that epigallocatechin-3-gallate has been shown to inhibit the growth of SV40 virally transformed W138 cells, without any observed effects against their normal counterparts. The IC_50_ value of epigallocatechin-3-gallate was estimated to be 120 and 10 µM for normal versus the transformed cells, respectively [27]. We have, therefore, proposed that the prooxidant activity of plant polyphenols, as opposed to their antioxidant property, is much more important for their observed and documented anticancer properties. In recent years, a number of independent research groups have confirmed our results and our hypothesis, which has resulted in increased acceptance of a prooxidant behavior of these compounds [28,29]. We have also shown the prooxidant DNA damaging effects of resveratrol in a mouse model with high copper concentrations [30]. These and other studies unequivocally support the conclusion that, in addition to an antioxidant behavior, natural dietary and diet-derived polyphenol compounds can elicit prooxidant behavior. We trust that this property cannot be ignored when planning a clinical trial with a focus on natural agents’ anticancer activity. It is critical to consider the relatively poor bioavailability of plant polyphenols that is a result of their efficient biotransformation and quick elimination. As an example, the highest plasma concentration of resveratrol (2.6 ± 1 µM) has been reported to be attained within the first five minutes after administration of 20 mg resveratrol/kg body weight orally [31]. Further, it should be noted that gallocatechins are only one class of polyphenol compounds, among several polyphenols consumed as part of the normal human diet. Since several other polyphenols present in diet, such as flavonoids and tannins are also actively prooxidant agents [13], their cumulative bioavailability, plasma concentration, anticancer effects, etc., should be much more than just a single polyphenol. As a proof, it has been shown that a combination of epigallocatechin-3-gallate with luteolin is more effective than either polyphenol alone, in inducing apoptosis in cancer cells in vitro as well as the inhibition of tumor growth in nude mouse xenograft model [32].

Cancer is a highly complex genetic disease, where even the identities of the various genes involved in a given cancer type are not yet known. It is believed that inherited genetic variations as well as the acquired genomic aberrations contribute to cancer initiation and progression. Recently, at least ten subgroups of breast cancer have been identified with different set of genes mutated in terms of copy number variants and single nucleotide polymorphisms in each subgroup [33]. Another study has recognized that a number of agents used for cancer therapy, such as ionizing radiations and the chemotherapeutic agents, function through the generation of ROS, resulting in the inhibition of cell cycle and induction of apoptosis [34]. Under these circumstances, it is highly unlikely to discover a single bullet cure for various cancers. Recently, the focus of cancer research has shifted to the biochemistry/metabolism of cancer cells. In this context, our findings assume considerable significance as they provide a rationale for the design of novel anticancer compounds based on targeting the elevated copper levels in cancer cells.

## 4. Materials and Methods

(−)-Epicatechin-3-gallate, tannic acid, cupric chloride, bathocuproine disulphonate, neocuproine, superoxide dismutase (SOD), agarose, Histopaque 1077, Triton X-100 and Trypan blue were purchased from Sigma (St. Louis, MO, USA). Epicatechin-3-gallate was always prepared fresh, as a 3 mM stock in double distilled water (ddH_2_O). Epicatechin-3-gallate was observed to always remain in solution, in all the reaction conditions. MDA-MB-468 breast cancer cells were maintained in RPMI medium (Invitrogen, Carlsbad, CA, USA), which was supplemented with 10% foetal bovine serum (FBS). Cancer cells were cultured at 37 °C in a 5% CO_2_-incubator.

### 4.1. Isolation of Lymphocytes

Heparinized blood samples (2 mL), from a single healthy donor, were obtained by venipuncture. They were diluted in Ca^2+^ and Mg^2+^ free PBS. Lymphocytes were isolated using Histopaque 1077 (Sigma), and the cells were finally cultured in RPMI medium.

### 4.2. Viability Assessment of Lymphocytes

The lymphocytes were checked for their viability, before the start and after the end of the reaction, using Trypan Blue Exclusion Test [35].

### 4.3. Treatment of Lymphocytes and Evaluation of DNA Breakage by Alkaline Single-Cell Gel Electrophoresis (Comet Assay)

Lymphocytes were obtained from a donor. They were diluted to a concentration of 2 × 10^5^ cells in 2 mL of RPMI medium. After counting, ~10,000 lymphocytes were added to warm low melting point agarose in PBS (75 µL). The mixture was immediately applied to a frosted microscopic slide that had a layer of standard agarose (1%) in PBS. Low melting agarose was allowed to harden for 10 min on the slides at 4 °C. Thereafter, the layered lymphocytes on slides were subjected to epicatechin-3-gallate treatment, and analyzed by Comet assay, as described previously [18]. Permeabilized lymphocytes were also treated with epicatechin-3-gallate in individual experiments [36]. For permeabilization, prior to treatment with epicatechin-3-gallate, lymphocytes were incubated in permeabilization solution (0.5% Triton X-100 in 0.004 M Tris-HCl, pH 7.4) for 10 min on ice. For treatment, each slide was transferred into a rectangular dish that also contained epicatechin-3-gallate and other additions, as identified for individual experiments. Slides were incubated at 37 °C for indicated time periods (1 h for intact cells/30 min for permeabilized cells) and then washed twice in 0.4 M phosphate buffer, pH 7.5, for 5 min at room temperature, before being subjected to Comet assay.

### 4.4. Treatment of pBR322 DNA

Reaction mixture (30 µL) containing 10 mM Tris-HCl (pH 7.5), 0.5 µg of plasmid DNA and epicatechin-3-gallate was incubated at 37 °C for 2 h. Then, 10 µL of a solution containing 40 mM EDTA, 0.05% bromophenol blue (tracking dye) and 50% (*v*/*v*) glycerol was added to the reaction mixture, and electrophoresis was performed using agarose gels (1%). Ethidium bromide (0.5 µg/mL) was used to stain the gels prior to visualization and imaging on a UV-transilluminator.

### 4.5. Measurement of Intracellular ROS

ROS generation was assessed in lymphocytes after exposure to epicatechin-3-gallate alone or in presence of Cu(II), using DCFH-DA dye as fluorescence agent [37]. Lymphocytes were pre-incubated with 50 µM Cu(II) for 1 h prior to addition of epicatechin-3-gallate for another 1 h at 37 °C. After exposure, cells were incubated with DCFH-DA (10 mM) for 30 min at 37 °C. Reaction mixture was then removed and cells washed twice in PBS. After excitation at 485 nm, fluorescence was recorded on a spectrofluorometer (Shimadzu, Japan) at 528 nm.

### 4.6. Detection of H_2_O_2_ in the Incubation Medium by FOX Assay

Quantification of H_2_O_2_, generated by epicatechin-3-gallate, was monitored by adapting FOX assay. The principle of the assay involved oxidation of ferrous (Fe^2+^) to ferric (Fe^3+^) ions by H_2_O_2_ which subsequently bonded with the xylenol orange dye in the incubation mixture containing RPMI and phosphate buffer 0.4 M (pH 7.5). The reaction mixture, containing epicatechin-3-gallate, was incubated at 37 °C for 2 h, and an aliquot of 200 µL was used to detect H_2_O_2_ [18]. The end point was the generation of an orange to purple complex that was measured at 560 nm.

### 4.7. Determination of TBARS

Thiobarbituric acid reactive substance was determined, using the method of Ramanathan et al. [38]. Cells (1 × 10^5^ mL) were incubated with indicated doses of epicatechin-3-gallate at 37 °C for 1 h, before being centrifuged at 1000 rpm. Cells were pre-incubated with fixed concentrations of neocuproine and/or thiourea for individual experiments, as identified in the figure legends. Cell pellet was washed in PBS twice before suspension in 0.1 N NaOH. Cell suspension (1.4 mL) was further exposed to 10% TCA and 0.6 M TBA (2-thiobarbituric acid) in boiling water bath for a total of 10 min. Absorbance was monitored at 532 nm. Molar extinction coefficient was used to calculate nanomoles of TBA reactive substance.

### 4.8. Cell Growth Inhibition as Studied by MTT Assay

MDA-MB-468 cells (1 × 10^4^ per well) were seeded in 96-well microtiter culture plates. After overnight incubation, media was replaced with fresh medium with or without indicated concentrations of epicatechin-3-gallate. After desired incubation time periods; MTT solution (from a 5 mg/mL stock solution in PBS) was added to each well and incubated further for 2 h at 37 °C. Upon completion of incubation with MTT solution, supernatant was aspirated and the MTT formazan, formed by metabolically viable cells, was dissolved in DMSO (100 µL) by mixing for 30 min on a gyratory shaker. Absorbance was measured on an Ultra Multifunctional Microplate Reader (Bio-Rad, Hercules, CA, USA).

### 4.9. Statistics

Results are expressed as mean ± SEM/SD and the statistical analysis was performed, as described previously by Tice et al. [39]. Student’s *t*-test or the Analysis of variance (ANOVA) was used, as appropriate, to examine the statistical significance. *p* < 0.05 was considered statistically significant.

## 5. Conclusions

Copper levels are known to be considerably elevated in almost all types of cancers. In this study, we show that green tea polyphenol epicatechin-3-gallate possesses anticancer activity and is able to target endogenous copper leading to pro-oxidant signaling and consequent cell death. We believe that such a mechanism explains the anticancer effect of epicatechin-3-gallate as also its preferential cytotoxicity towards cancer cells. This work can possibly be utilized for the synthesis and development of novel anticancer drugs with greater cytotoxicity and bioavailability.

## Figures and Tables

**Table 1:** Effect of reactive oxygen species scavengers on epicatechin-3-gallate induced cellular DNA breakage in permeabilized cells.

Dose	Comet Tail Length (µm)	% Inhibition of Tail Length (ECG Alone − Scavenger)/ECG Alone × 100
Control (untreated)	2.67 ± 0.18	-
Epicatechin-3-gallate *(*50 µM)	22.91 ± 0.81 #	-
+SOD (100 µg/mL)	6.19 ± 0.46 *	72.98
+Catalase (100 µg/mL)	9.01 ± 0.63 *	60.67
+Thiourea (1 mM)	8.23 ± 0.68 *	64.07

* *p <* 0.05, compared with control cells (# epicatechin-3-gallate treatment alone).

**Table 2:** Effect of various metal chelators on cellular DNA degradation in lymphocytes induced by epicatechin-3-gallate.

Dose	Permeabilized Cells		Intact Cells	
	Comet Tail Length (µm)	% of Control	Comet Tail Length (µm)	% of Control
Control	2.83 ± 0.19 ^1^	-	2.45 ± 0.15 ^1^	-
Epicatechin-3-gallate (50 µM)	13.17 ± 0.79 ^2^	-	22.16 ± 1.56 ^3^	-
+Neocuprione (50 µM)	8.24 ± 0.21 ^3^	37.43	10.48 ± 0.31 ^3^	52.70
+Bathocuprione (50 µM)	8.96 ± 0.35 ^3^	31.96	20.54 ± 1.04 ^3^	7.31
+Hisitidine (50 µM)	12.60 ± 0.66 ^3^	4.32	21.31 ± 1.52 ^3^	3.83
+Desferioxamine mesylate (50 µM)	12.67 ± 0.68 ^3^	3.79	20.78 ± 1.49 ^3^	6.22

^1^ The values shown represent epicatechin-3-gallate induced DNA breakage in intact and permeabilized cells in the presence of metal chelators measured as a percentage of the control (DNA breakage induced by epicatechin-3-gallate in the absence of any chelator); ^2^ *p <* 0.05 when compared with ^1^, within the respective group (permeabilized/intact cells); ^3^ *p <* 0.05 when compared with ^2^, within the respective group (permeabilized/intact cells). In both cases, percentage of tail length inhibition (% of control) has been calculated using formula: (ECG alone − Metal chelator)/ECG alone × 100.

## Supplementary Materials

Supplementary materials can be found at www.mdpi.com/1422-0067/18/1/34/s1.

## References

[1] Hanasaki Y., Ogawa S., Fukui S. (1994). The correlation between active oxygens scavenging and antioxidative effects of flavonoids. Free Radic. Biol. Med..

[2] Mukhtar H., Das M., Khan W.A., Wang Z.Y., Bik D.P., Bickers D.R. (1988). Exceptional activity of tannic acid among naturally occurring plant phenols in protecting against 7,12-dimethylbenz(a)anthracene-, benzo(a)pyrene-, 3-methylcholanthrene-, and N-methyl-N-nitrosourea-induced skin tumorigenesis in mice. Cancer Res..

[3] Inoue M., Suzuki R., Koide T., Sakaguchi N., Ogihara Y., Yabu Y. (1994). Antioxidant, gallic acid, induces apoptosis in HL-60RG cells. Biochem. Biophys. Res. Commun..

[4] Ahmad N., Feyes D.K., Nieminen A.L., Agarwal R., Mukhtar H. (1997). Green tea constituent epigallocatechin-3-gallate and induction of apoptosis and cell cycle arrest in human carcinoma cells. J. Natl. Cancer Inst..

[5] Kuo M.L., Huang T.S., Lin J.K. (1996). Curcumin, an antioxidant and antitumor promoter, induces apoptosis in human leukemia cells. Biochim. Biophys. Acta.

[6] Clement M.V., Hirpara J.L., Chawdhury S.H., Pervaiz S. (1998). Chemopreventive agent resveratrol, a natural product derived from grapes, triggers CD95 signaling-dependent apoptosis in human tumor cells. Blood.

[7] Said Ahmad M., Fazal F., Rahman A., Hadi S.M., Parish J.H. (1992). Activities of flavonoids for the cleavage of DNA in the presence of Cu(II): Correlation with generation of active oxygen species. Carcinogenesis.

[8] Khan N.S., Hadi S.M. (1998). Structural features of tannic acid important for DNA degradation in the presence of Cu(II). Mutagenesis.

[9] Ahsan H., Hadi S.M. (1998). Strand scission in DNA induced by curcumin in the presence of Cu(II). Cancer Lett..

[10] Malik A., Azam S., Hadi N., Hadi S.M. (2003). DNA degradation by water extract of green tea in the presence of copper ions: Implications for anticancer properties. Phytotherapyresearch.

[11] Ahmad A., FarhanAsad S., Singh S., Hadi S.M. (2000). DNA breakage by resveratrol and Cu(II): Reaction mechanism and bacteriophage inactivation. Cancer Lett..

[12] Kagawa T.F., Geierstanger B.H., Wang A.H., Ho P.S. (1991). Covalent modification of guanine bases in double-stranded DNA. The 1.2-A Z-DNA structure of d(CGCGCG) in the presence of CuCl_2_. J. Biol. Chem..

[13] Hadi S.M., Asad S.F., Singh S., Ahmad A. (2000). Putative mechanism for anticancer and apoptosis-inducing properties of plant-derived polyphenolic compounds. IUBMB Life.

[14] Schumacker P.T. (2006). Reactive oxygen species in cancer cells: Live by the sword, die by the sword. Cancer Cell.

[15] Trachootham D., Zhou Y., Zhang H., Demizu Y., Chen Z., Pelicano H., Chiao P.J., Achanta G., Arlinghaus R.B., Liu J. (2006). Selective killing of oncogenically transformed cells through a ROS-mediated mechanism by β-phenylethylisothiocyanate. Cancer Cell.

[16] Azmi A.S., Bhat S.H., Hadi S.M. (2005). Resveratrol-Cu(II) induced DNA breakage in human peripheral lymphocytes: Implications for anticancer properties. FEBS Lett..

[17] Ostling O., Johanson K.J. (1984). Microelectrophoretic study of radiation-induced DNA damages in individual mammalian cells. Biochem. Biophys. Res. Commun..

[18] Shamim U., Hanif S., Ullah M.F., Azmi A.S., Bhat S.H., Hadi S.M. (2008). Plant polyphenols mobilize nuclear copper in human peripheral lymphocytes leading to oxidatively generated DNA breakage: Implications for an anticancer mechanism. Free Radic. Res..

[19] Azmi A.S., Bhat S.H., Hanif S., Hadi S.M. (2006). Plant polyphenols mobilize endogenous copper in human peripheral lymphocytes leading to oxidative DNA breakage: A putative mechanism for anticancer properties. FEBS Lett..

[20] Badwey J.A., Karnovsky M.L. (1980). Active oxygen species and the functions of phagocytic leukocytes. Annu. Rev. Biochem..

[21] Long L.H., Clement M.V., Halliwell B. (2000). Artifacts in cell culture: Rapid generation of hydrogen peroxide on addition of (−)-epigallocatechin, (−)-epigallocatechingallate, (+)-catechin, and quercetin to commonly used cell culture media. Biochem. Biophys. Res. Commun..

[22] Bhat R., Hadi S.M. (1994). DNA breakage by tannic acid and Cu(II): Sequence specificity of the reaction and involvement of active oxygen species. Mutat. Res..

[23] Smith C., Halliwell B., Aruoma O.I. (1992). Protection by albumin against the pro-oxidant actions of phenolic dietary components. Food Chem. Toxicol..

[24] Quinlan G.J., Gutteridge J.M. (1987). Oxygen radical damage to DNA by rifamycin SV and copper ions. Biochem. Pharmacol..

[25] Khan H.Y., Zubair H., Faisal M., Ullah M.F., Farhan M., Sarkar F.H., Ahmad A., Hadi S.M. (2014). Plant polyphenol induced cell death in human cancer cells involves mobilization of intracellular copper ions and reactive oxygen species generation: A mechanism for cancer chemopreventive action. Mol. Nutr. Food Res..

[26] Raffoul J.J., Kucuk O., Sarkar F.H., Hillman G.G. (2012). Dietary agents in cancer chemoprevention and treatment. J. Oncol..

[27] Chen Z.P., Schell J.B., Ho C.T., Chen K.Y. (1998). Green tea epigallocatechin gallate shows a pronounced growth inhibitory effect on cancerous cells but not on their normal counterparts. Cancer Lett..

[28] Michels G., Watjen W., Weber N., Niering P., Chovolou Y., Kampkotter A., Proksch P., Kahl R. (2006). Resveratrol induces apoptotic cell death in rat H4IIE hepatoma cells but necrosis in C6 glioma cells. Toxicology.

[29] Tyagi A., Gu M., Takahata T., Frederick B., Agarwal C., Siriwardana S., Agarwal R., Sclafani R.A. (2011). Resveratrol selectively induces DNA damage, independent of Smad4 expression, in its efficacy against human head and neck squamous cell carcinoma. Clin. Cancer Res..

[30] Ullah M.F., Ahmad A., Zubair H., Khan H.Y., Wang Z., Sarkar F.H., Hadi S.M. (2011). Soy isoflavone genistein induces cell death in breast cancer cells through mobilization of endogenous copper ions and generation of reactive oxygen species. Mol. Nutr. Food Res..

[31] Asensi M., Medina I., Ortega A., Carretero J., Bano M.C., Obrador E., Estrela J.M. (2002). Inhibition of cancer growth by resveratrol is related to its low bioavailability. Free Radic. Biol. Med..

[32] Amin A.R., Wang D., Zhang H., Peng S., Shin H.J., Brandes J.C., Tighiouart M., Khuri F.R., Chen Z.G., Shin D.M. (2010). Enhanced anti-tumor activity by the combination of the natural compounds (−)-epigallocatechin-3-gallate and luteolin: Potential role of p53. J. Biol. Chem..

[33] Curtis C., Shah S.P., Chin S.F., Turashvili G., Rueda O.M., Dunning M.J., Speed D., Lynch A.G., Samarajiwa S., Yuan Y. (2012). The genomic and transcriptomic architecture of 2000 breast tumours reveals novel subgroups. Nature.

[34] Watson J. (2013). Oxidants, antioxidants and the current incurability of metastatic cancers. Open Boil..

[35] Pool-Zobel B.L., Guigas C., Klein R., Neudecker C., Renner H.W., Schmezer P. (1993). Assessment of genotoxic effects by lindane. Food Chem. Toxicol..

[36] Czene S., Tiback M., Harms-Ringdahl M. (1997). pH-dependent DNA cleavage in permeabilized human fibroblasts. Biochem. J..

[37] Wang H., Joseph J.A. (1999). Quantifying cellular oxidative stress by dichlorofluorescein assay using microplate reader. Free Radic. Biol. Med..

[38] Ramanathan R., Das N.P., Tan C.H. (1994). Effects of gamma-linolenic acid, flavonoids, and vitamins on cytotoxicity and lipid peroxidation. Free Radic. Biol. Med..

[39] Tice R.R., Strauss G.H. (1995). The single cell gel electrophoresis/comet assay: A potential tool for detecting radiation-induced DNA damage in humans. Stem Cells.

