# Supplementary material for: Mobilization of Nuclear Copper by Green Tea Polyphenol Epicatechin-3-Gallate and Subsequent Prooxidant Breakage of Cellular DNA: Implications for Cancer Chemotherapy
Source: Int J Mol Sci. 2016 Dec 26;18(1):34. doi: 10.3390/ijms18010034 (PMC5297669; doi:10.3390/ijms18010034)
Supplement: Supplementary file 1 [file ijms-18-00034-s001.pdf]

Supplementary Materials: Mobilization of Nuclear Copper by Green Tea Polyphenol Epicatechin-3-Gallate and Subsequent Prooxidant Breakage of Cellular DNA: Implications for Cancer Chemotherapy

Mohd Farhan, Mohammad Oves, Sandesh Chibber, Sheikh Mumtaz Hadi and Aamir Ahmad

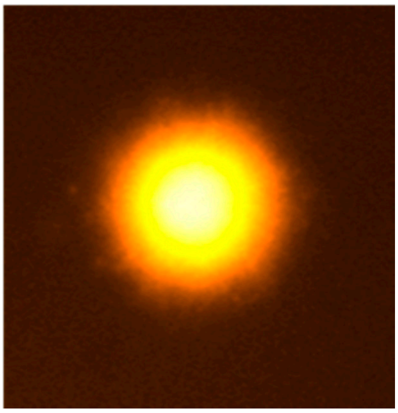

(**A**)

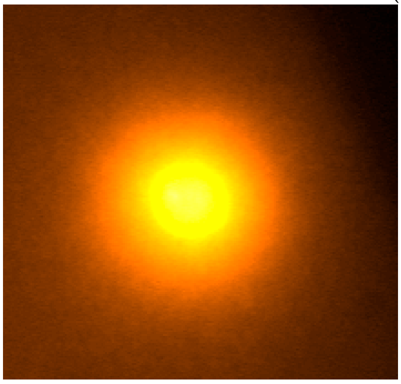

(**B**)

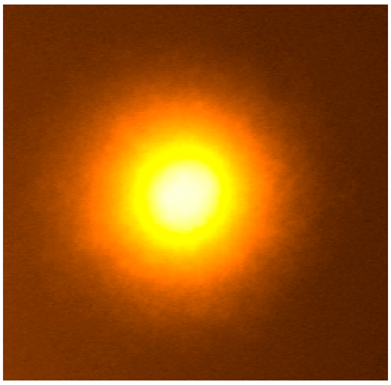

(**C**)

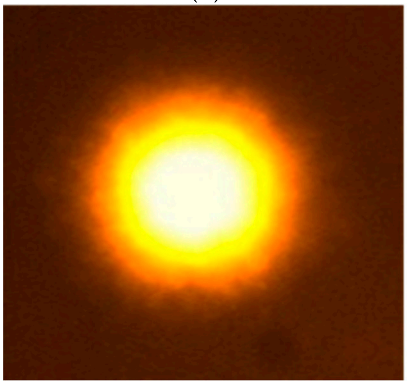

(**D**)

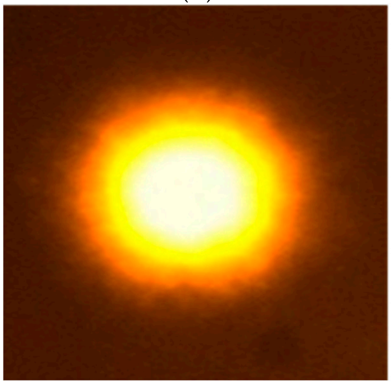

(**E**)

Figure S1. *Cont.*

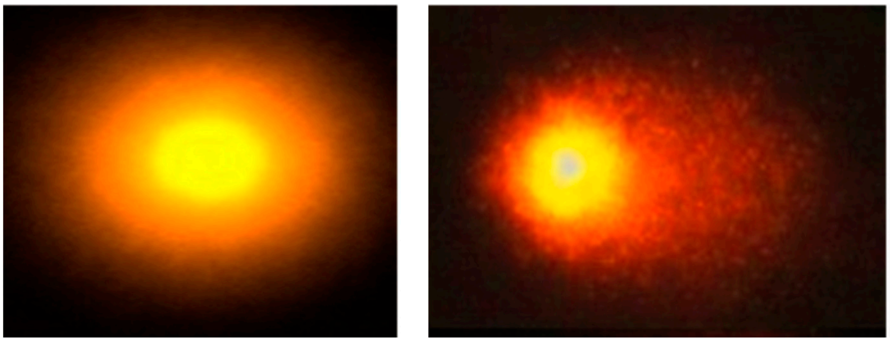

(**F**) (**G**)

Figure S1. Single cell gel electrophoresis of human peripheral lymphocytes showing Comets (100×) after treatment with different concentrations of Epicatechin-3-gallate and Cu(II): (**A**) Untreated; (**B**) ECG alone (10 μM); (**C**) ECG (10 μM) + Cu(II) 50 μM; (**D**) ECG alone (25 μM); (**E**) ECG (25 μM) + Cu(II) (50 μM); (**F**) ECG alone (50 μM); (**G**) ECG (50 μM) + Cu(II) (50 μM).
